# Improving preparedness for time critical prehospital care: a descriptive study of the first responder system in Central Norway

**DOI:** 10.1186/s13049-024-01316-9

**Published:** 2025-01-25

**Authors:** Andreas Lindeman, Lars Eide Næss, Lars Vesterhus, Ann-Britt Maude Bakken, Andreas Krüger, Helge Haugland

**Affiliations:** 1 Faculty of Medicine and Health Sciences, Norwegian University of Science and Technology (NTNU), 7018 Trondheim, Norway; 2 Department of Emergency Medicine and Pre-Hospital Services, St. Olav’s University Hospital, Trondheim, Norway; 3 Department of Research and Development, Norwegian Air Ambulance Foundation, Oslo, Norway

**Keywords:** First responders, Emergency medical services, Health care, Descriptive studies, Health care preparedness, Quality of care

## Abstract

**Background:**

First responders exist in several countries and have been a prehospital emergency medical resource in Norwegian municipalities since 2010. However, the Norwegian system has not yet been studied. The aim of this study was to describe the first responder system in Central Norway and how it is used as a supplement to emergency medical services (EMS).

**Methods:**

We described incidents with dispatch of first responders in the catchment area of the Emergency Medical Communication Center of Sør-Trøndelag in Central Norway, using retrospective data recorded in the Norwegian Emergency Medical Information System between 2019 and 2023.

**Results:**

First responders were dispatched to 460 incidents during the period. Of these, 441/460 (96%) incidents were assessed as “acute”, and 135/460 (29%) were assessed as possible cardiac arrests. Four large rural municipalities accounted for 234/460 (51%) of the incidents. One in four patients, 112/449 (25%), died within 30 days. EMS had a median response time of 29 min in our sample.

**Conclusion:**

First responders are almost exclusively dispatched to high-severity incidents, with suspected cardiac arrest being the most common dispatch criteria. Our findings suggest that the first responder system contributes to rapid response in cases of acute illness and injury, especially in rural areas.

## Background

The European Resuscitation Council (ERC) has identified five conditions that particularly depend on a well-functioning and accessible prehospital emergency medical service (EMS). These five conditions, known as the "First Hour Quintet," include cardiac arrest, respiratory failure, severe trauma, chest pain, and stroke. Common to these are their high mortality and morbidity, and that beneficial outcomes largely depend on early prehospital assessment and treatment, as well as rapid transport to a higher level of care [1].

Due to large, but sparsely populated areas, establishing equitable access to EMS in Norway is difficult, in turn making it challenging to meet the short treatment windows required for these conditions [2]. Internationally, several countries employ first responder systems to mitigate challenges in EMS response [3–16]. These systems involve personnel from the fire and rescue services (FRS), police officers, or laypeople in the role as first responders, who are usually trained in cardiopulmonary resuscitation with defibrillation (CPR-D). Research on these systems is mostly descriptive, and several studies have concluded that first responders might shorten response times, time to CPR-D, and increase ROSC rates and survival in out-of-hospital cardiac arrest (OHCA) [3–9, 11–15, 17]. There are also qualitative studies describing ethical issues and experiences with the use of first responders [10].

The Norwegian national first responder system was initiated as part of multiple projects to increase society's preparedness for time-critical conditions such as cardiac arrest, stroke, myocardial infarction, and severe trauma. The Norwegian first responders are personnel employed in the FRS who have received training in first aid and CPR-D and are able to support the first aid effort in time-critical medical emergencies until the first professional healthcare provider arrives. The purpose of the system is to supplement existing EMS [18].

Thus, the first responder system represents a potentially critical resource. However, since its establishment in 2010, no studies have described the operational and clinical activity of the system in Norway. This study investigates the role of first responders as an adjunct to prehospital emergency medical care in Central Norway. It attempts to answer how often first responders are used, describe the patient population including patient outcomes, and compare the response times of first responders with those of EMS in the catchment area of Sør-Trøndelag Emergency Medical Communication Center (EMCC) in Central Norway between 2019 and 2023.

## Methods

This was a retrospective observational study. The article was planned and conducted according to the Strengthening the Reporting of Observational studies in Epidemiology (STROBE) guidelines recommended by the Equator Network [19]. The study was submitted for review to the Regional Committee for Medical and Health Research Ethics in Central Norway (REK Midt). Being assessed as quality improvement work, the study was not subject to mandatory submission to REK (Application number 581902).

### Study setting

**Fig. 1:**
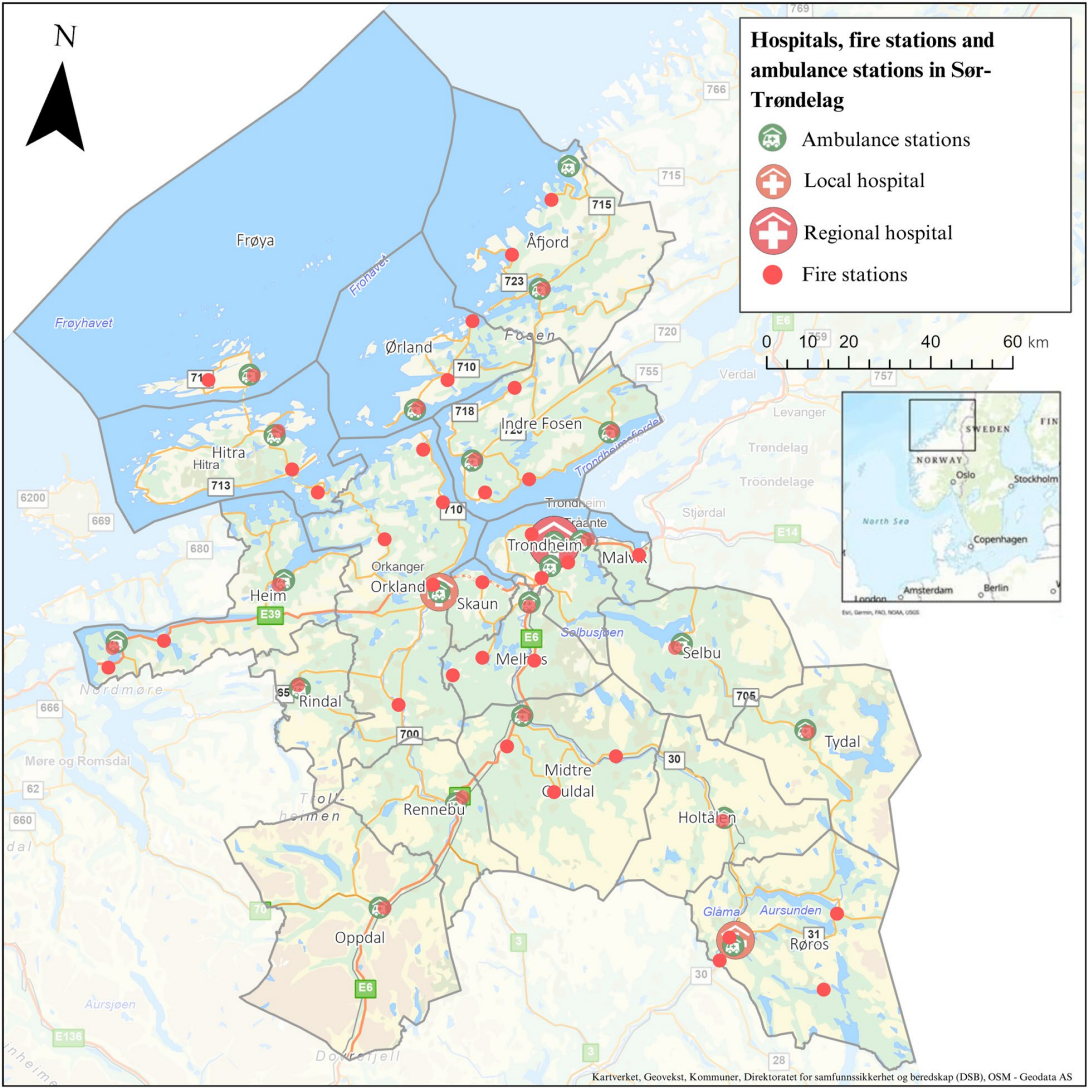
Hospitals, ambulance stations, and fire stations in the coverage area of Sør-Trøndelag EMCC, 2024. The background image is sourced from www.geodata.no (Esri, Kartverket, Geovekst, Municipalities, OSM, USGS, Garmin, FAO, NOAA, DSB), with permission from Geodata AS

The study was conducted within the catchment area of the Sør-Trøndelag EMCC, covering 21 municipalities with a total population of 320,884 across an area of 18,848 square kilometres. This region comprises 22 ambulance stations, 50 fire stations and both a regional university hospital, as well as a local hospital, as depicted in Fig. 1 [20].

The FRS is the most decentralized emergency service in Norway, with more than 12,000 personnel and 600 fire stations located in nearly every municipality (2020). In 2023, over 8200 FRS personnel, distributed across 330 fire stations, were active first responders [21].

First responders on-call are visible in the EMCC´s map of available resources. Dispatching first responders is considered in patients suffering from illness or injury involving possible or confirmed failure of vital functions, where first responders are likely to arrive earlier than EMS [18]. The EMCC requests assistance from first responders by contacting the Fire and Rescue Dispatch Centre (FRDC). First responders are specifically trained and intended to initiate life-saving measures in cases of cardiac arrest, unconsciousness, severe trauma, and risk of hypothermia [18]. Deployment of first responders varies by municipality: some personnel remain on-call at fire stations, while others are permitted to respond from home, provided that it is within close proximity of the station. Alerts are transmitted via personal radios, and they respond in fire trucks or other service vehicles. First responders are equipped with automated external defibrillators (AEDs), ventilation masks, and equipment for haemorrhage control and hypothermia prevention. Additionally, they are authorized to administer oxygen if the municipal physician provides the requisite training [18]. First responders are not required to document their findings and the treatment initiated but should report this when EMS arrives.

The first responder system is regulated according to the "National First Responder Guideline" issued by the Norwegian Directorate of Health [18]. The Norwegian Air Ambulance Foundation (NAAF) provides a standardized 8-h training course certifying first responders in accordance with the guideline.

### Data collection

We included all incidents from the EMCC during the period from 2019 to 2023 where first responders were dispatched. Data were retrieved from the Emergency Medical Information System (AMIS, OMDA AS, Oslo, Norway), an IT support tool used by Norwegian EMCC´s. Time of death was retrieved from the Norwegian Cause of Death Registry via the Patient Administrative System (PAS) of the Central Norway Regional Health Authority (Helse-Midt RHF).

We extracted data from the Emergency Medical Information System that could be used to describe the incidents where first responders were dispatched. This included the number of incidents, urgency level of the incidents (acute, urgent or normal response**)**, municipality, Ground Emergency Medical Services (GEMS) and Helicopter Emergency Medical Service (HEMS) involvement, other resources involved (general practitioners, rescue helicopters, home care nurses, police officers and personnel from voluntary rescue organizations), patient age and sex, mortality and specialized medical teams assembled on patient admission to the hospital (medical emergency team, trauma team and stroke teams)**.** The prehospital index groups of the patients, which are categorizations based on the patient's signs and symptoms according to the Norwegian Index for Emergency Medical Assistance [22], were obtained from the same data. An example of such an index group is "Unresponsive adult – not breathing normally", which indicate suspected cardiac arrest, in accordance with the definition from the European Resuscitation Council (ERC) [17]. The median response time, defined as the time from when a resource is alarmed by a dispatcher to when it arrives, was included for GEMS and HEMS if they were dispatched. The response times were compared with the median response times for FRS on health-related missions in the same municipalities from 2019 to 2023, provided by the Norwegian Directorate for Civil Protection (DSB) who in turn obtain this information from the FRDC. Data from the National Cardiac Arrest Registry has been examined to assess the role of first responders who are dispatched to cardiac arrests in Central Norway during the same period.

### Data analysis

We used IBM SPSS Statistics 27 (IBM Corporation, Armonk, NY, USA) for data analysis. Continuous data that were not normally distributed are presented as the median and interquartile range (IQR). Categorical data are presented as numbers and percentages.

## Results

During the period from 2019 to 2023, first responders were dispatched to 460 incidents. In comparison, HEMS was involved in 4,314 incidents, and GEMS were dispatched to 168,350 incidents.

**Fig. 2:**
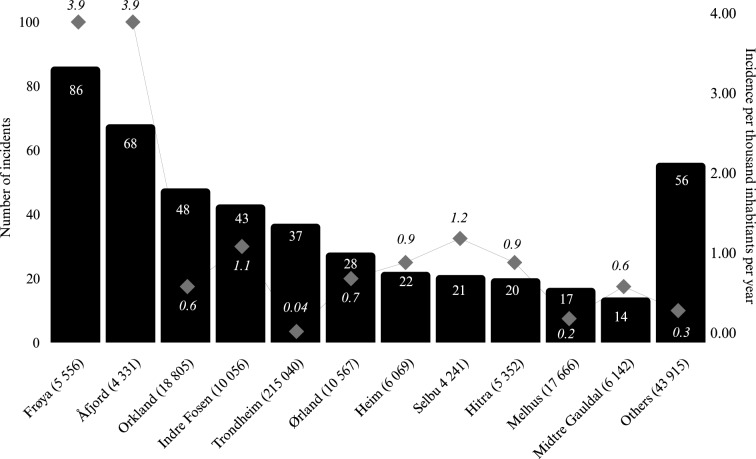
Overview of municipalities (with number of inhabitants in parenthesis) in Sør-Trøndelag in Central Norway with the highest number of first responder dispatches. The gray boxes indicate incidence of dispatches per thousand inhabitants per year in each municipality

Two rural municipalities (Frøya and Åfjord) accounted for the largest proportion of incidents where first responders were dispatched, with 86/460 (19%) and 68/460 (15%) incidents respectively, whereas the more densely populated city of Trondheim accounted for 37/460 (8%) incidents. Incidents where first responders were dispatched compared with the incidence of first responder dispatches per thousand inhabitants per year is shown in Fig. 2*.*

### EMS cooperation

Multiple resources may be involved in a single incident. In 441/460 (96%) of the first responder incidents, GEMS was also dispatched, while HEMS was involved in 206/460 (45%) of the first responder incidents. In 213/460 (46%) incidents where first responders were dispatched, other resources besides EMS were involved (general practitioners, rescue helicopters, home care nurses, police officers and personnel from voluntary organizations).

In 19/460 (4%) incidents, first responders were dispatched without GEMS, and out of these, 12/19 (63%) incidents occurred on one particular island municipality (Frøya). In 18 of the 19 incidents, a healthcare provider other than GEMS arrived at the scene, whereas one incident was handled solely by the first responders.

The urgency level for the incidents where first responders were dispatched was classified as "acute" in 443/460 (96%) of the cases, whereas the remaining 17/460 (4%) were marked as "urgent".

### Patients and clinical characteristics

Of the 460 missions involving first responders, 131/460 (28%) were categorized as "Unresponsive adult – not breathing normally", and 4/460 (1%) as "Unresponsive child – not breathing normally". Chest pain and breathing difficulties were the second most common categories, each accounting for 11% of the missions (Table 1).

**Table 1:** Common index groups

Prehospital Index Group	Number (%)
Unresponsive adult – not breathing normally	131 (28%)
Chest pain	52 (11%)
Breathing problems	52 (11%)
Neurology (seizures, altered level of consciousness)	43 (9%)
Serious condition/Unstable vital signs/Suspected red criteria	39 (8%)
Trauma	37 (8%)
Affected circulation (pale, clammy)	26 (6%)
Possible stroke	23 (5%)
Intoxication and mental health problems	10 (2%)
Abdominal pain	9 (2%)
Unresponsive child – not breathing normally	4 (1%)
Other	34 (7%)
Total	460 (100%)

Grouped overview of the most used index groups from the Norwegian Index for Emergency Medical Assistance version 4.2 for incidents where first responders were dispatched. Related index groups are combined

Among the patients cared for by the first responders, 280/460 (61%) were men and 284/460 (62%) were between 60 and 89 years old (Table 2).

**Table 2:** Age distribution of patients for whom first responders were dispatched

Age	Number of patients, (%)
0–9	14 (3%)
10–19	12 (3%)
20–29	16 (4%)
30–39	22 (5%)
40–49	33 (7%)
50–59	63 (14%)
60–69	92 (21%)
70–79	116 (26%)
80–89	76 (17%)
90–99	13 (3%)
100 +	0 (0%)
Missing data	3 (1%)

Mortality data were missing for 11/460 (2%) of the patients. A total of 94/449 (21%) of the patients died before arriving at a hospital, 7/449 (2%) died within the first day in the hospital, and an additional 11/449 (2%) died within 30 days of the incident.

Among the patients admitted to the hospital, 52/355 (15%) were assessed by some kind of specialized medical team. Of these, a medical emergency team (MET) handled 23/355 (7%) of the patients, whereas trauma and stroke teams handled 17/355 (5%) and 12/355 (3%) of the patients, respectively.

In our sample, GEMS had a median response time of 29 min and 10 s (IQR: 17 min 58 s–39 min 7 s), and HEMS had a median response time of 28 min and 22 s (IQR: 23 min 26 s–37 min 1 s).

## Discussion

First responders were dispatched to 460 medical emergencies in the Central Norwegian municipalities of Sør-Trøndelag from 2019 to 2023. They primarily responded to patients with suspected time-critical conditions, that had high prehospital mortality rates. EMS response times to the same incidents were long.

The patient population assisted by first responders in Central Norway had a wide range of serious medical conditions, of which some are beyond the intended use of first responders according to the guidelines for the first responder system; i.e. cardiac arrest, unconsciousness, serious injuries, and risk of hypothermia [18]. One could discuss whether first responders should be used in conditions other than these pre-defined conditions, and if they should receive training, equipment, and possibly even medications to handle the patient population that they actually encounter. In a qualitative study related to Danish first responders by Sørensen et al. from 2023, the interviewees, who were paramedics and anaesthesiologists, saw the value of using first responders in cases of cardiac arrest but not in other medical emergencies [10].

In our study, the index group "Unresponsive adult—not breathing normally" was the largest single group, accounting for 28% of their dispatches. By comparison, this category accounted for 1% of the total GEMS dispatches during the period and 19% of the total HEMS alerts. This makes suspected cardiac arrest the most common cause for dispatching first responders. In some cases, first responders might reduce the time to CPR-D, which is documented to increase the survival after cardiac arrest [17]. Data from the Norwegian Cardiac Arrest Registry concerning cardiac arrest patients in Central Norway from 2019 to 2023, revealed that among 1236 patients with cardiac arrest where CPR was initiated, first responders were the first to attach a defibrillator in 128 out of 1236 (10%) cases [23].

First responders were more frequently used in rural municipalities compared to urban municipalities. One explanation might be that the time-saving benefit of dispatching first responders is smaller in cities, where GEMS usually are readily available or can be diverted. However, in municipalities with long distances and limited prehospital resources, the potential for saving time is greater, especially when multiple emergencies occur simultaneously [2, 7, 11]. Four of the 25 municipalities alone accounted for 51% of the incidents. These municipalities have a total of seven ambulances covering an area of 4.572 square kilometres with a population of 38,748 people. In municipalities such as these, it is plausible that first responders can provide a significant time advantage for patients, especially if EMS are busy.

The response times for first responders were not recorded in the Emergency Medical Information System such as those for EMS. Therefore, we obtained the median response time for FRS on health-related tasks (other than carrying and lifting) from 2019 to 2023 in the same Central Norwegian municipalities from statistics provided by DSB [24]. The data reveal that the FRS had a median response time of 13 min and 36 s for health-related missions during the same period [24]. As stated in the results, GEMS and HEMS in our sample had median response times of 29 min and 10 s, and 28 min and 22 s, respectively. Unfortunately, the data from DSB do not specify whether the FRS personnel were dispatched as first responders or not, thus the observed number of incidents from DSB differ somewhat from our own. However, the data can still provide a good indication of potential time savings by dispatching first responders, which is consistent with international literature on similar systems [6, 7, 9, 11–13].

The first responder system in Norway illustrates strategic use of decentralized resources to supplement, but not replace, existing EMS. This is especially important when factors such as long distances, multiple simultaneous emergencies, and limited resources can delay the treatment of critically ill patients [1, 2, 13]. However, certain policy questions are raised by the system. The use of fire and rescue personnel for health tasks could, in theory, affect fire preparedness in municipalities, especially when medical emergencies overlap with fire incidents. Furthermore, concern has also been raised as the FRS has been given responsibility related to emergency medical preparedness, and that the system's cost-saving potential could be used as an argument for deprioritizing GEMS response or downsizing GEMS resources in rural areas. The healthcare system in Norway, as in many other countries, faces resource constraints, and new approaches are sought after. One might question whether the first responder system could serve as a model for creating other innovative models of decentralized care or be expanded to involve other resources in municipalities, such as home care workers. There are strong indications that first responders are being used as an important adjunct for the treatment of urgent critical illness outside hospitals(5–7, 9, 13).

### Strengths and limitations

The study uses large data resources from the Emergency Medical Information System, while also integrating data from PAS, the Norwegian Cardiac Arrest Registry and DSB to provide actionable insights on the use of first responders. FRS exist in most countries, and we believe that our findings from both urban and rural areas in Central Norway are generalizable and relevant for similar systems abroad. Certain limitations need mentioning. The first being that the data are observational and based on both automatic data storage and manual recording, with the potential for incomplete documentation. Secondly, the study was not designed to assess the medical treatment initiated by the first responders, or to examine patient outcomes after first responder treatment. Thus, the study cannot provide decisive conclusions regarding the effect of first responders as a supplement to prehospital services. Examining the effects of the system could be relevant for future studies. However, data collection might prove challenging, as the National First Responder Guidelines do not require first responders to document their findings and initiated treatment but rather report this information to arriving EMS.

## Conclusion

First responders are almost exclusively dispatched to high-severity incidents, with suspected cardiac arrest being the most frequent dispatch criteria. As part of the most decentralized emergency service in Norway, first responders contribute to rapid response to critically ill patients – especially in areas with long EMS response times.

## Data Availability

The datasets used and/or analysed during the study are available from the corresponding author on reasonable request.

## Abbreviations

AED: Automated external defibrillator
CPR: Cardiopulmonary resuscitation
CPR-D: Cardiopulmonary resuscitation with defibrillation
DSB: The Norwegian Directorate for Civil Protection
EMCC: Emergency Medical Communication Centre
EMS: Emergency medical services
ERC: European Resuscitation Council
FRDC: Fire and Rescue Dispatch Centre
FRS: Fire and Rescue Service
NAAF: Norwegian Air Ambulance Foundation
OHCA: Out-of-hospital cardiac arrest
PAS: Patient administrative system
ROSC: Return of spontaneous circulation
